# The Growth and Scope of Voluntary Hospitals in London

**Published:** 1897-09-04

**Authors:** 


### The Institutional Workshop.

### THE GROWTH AND SCOPE OF VOLUNTARY HOSPITALS IN LONDON.

From a Correspondent.

### VII.—THE EXTENSION OF FREE DISPENSARIES.

Some institutions are so admirably framed to meet the needs of the people that they have only to be exhibited in working order to meet with prompt support and imitation. Such was the General Dispensary of which we have traced the rise. It soon became evident that under the dispensary system many more patients could be relieved at a trifling cost than could be received in all the hospitals in London with their vast revenues. And, though nobody ventured publicly to discuss the grave drawbacks to hospital treatment at that period, there were many who watched with anxiety the special symptoms seldom absent from the wards, and reluctantly admitted that "in places where a multitude of our species is confined at one time, diseases in general put on a sameness of appearance, and have all more or less a putrid tendency." To cure disease without exposing the poor to the dangers of residence in a hospital was one of the great aims of the promoters of the first dispensaries, and although surgical practice was not at first considered possible in the home of the patient, it was not long before this also was found to lie within the scope of the dispensary. In 1774 Westminster, always prominent in charities of this nature, founded a general dispensary in Gerrard Street, and added a surgical as well as a midwifery branch to the plan of the undertaking. They were soon able to prove that "the worst operations may be conducted at the homes of the patients with ease and with success," and their example was followed by the earlier and by all subsequent dispensaries.

The London Dispensary was founded three years later in Artillery Street, not far from the London Hospital. It was intended to serve the struggling population in the manufacturing districts of the East End, and embraced the parishes of Whitechapel, Shoreditch, Spitalfields, Wapping, and the adjacent riverside district. The stifled condition of Whitechapel at this period may be gathered from the fact that the parish of St. Mary's contained but 20 streets, the remainder consisting of 5 lanes, 29 alleys, 28 courts, 23 yards, and several other denominations of passages. The London Dispensary was a valuable adjunct to the work of the London Hospital, and appears to have been from the commencement very popular among the poor. Midwifery was not included in its scope.

The same year—1777—saw the establishment of a dispensary in Southwark for the benefit of the rapidly increasing population on the Surrey side of the river. It was situated within half a mile of Guy's and St. Thomas's, both of which hospitals had out-patients' departments, and as less than half the number of cases treated in the new dispensary, even including midwifery, were attended in their own homes, it would seem that we have here an early instance of overlapping in medical charity. As usual, however, the demand for free treatment was considerably in advance of the supply, and it must be remembered that hospital out-patients at this time were largely made up of former in-patients still requiring attention, and of persons awaiting admission.

The Metropolitan Dispensary was founded in 1779.

In 1780 the Finsbury Dispensary was opened, and is the first of which we learn that it was warmly supported by the parochial clergy. In spite of the fact that the ground covered was identical with the area occupied by the General Dispensary, the doctors had soon more work on their hands than they could get through. Their district included, besides several of the City parishes, Islington, St. Pancras, Holborn, and Bloomsbury. It would seem that the management did not command universal confidence, for it became necessary to frame a regulation that "no gratuity can be given to any officer without notice of the meeting for that purpose, inserted three times in the *Daily Advertiser*." A bad custom prevailed in some of these medical charities of rushing the election of new officials by the indiscriminate admission of the candidates' friends as new governors the day before the ballot, on payment of their subscription, purely in order to purchase a vote. The abuse was tacitly permitted by many institutions as bringing grist to the mill; but a more glaring example than usual of the process led a few years later to the founding of the rival New Finsbury Dispensary close by. Personal reasons were not wanting, and the dispensary was openly intended to "bring forward a truly shining character that might otherwise have remained unnoticed." The truly shining character, it may be divined, was Dr. Saunders, the physician extraordinary, of whom more will be heard. The New Finsbury Dispensary, though it seems to have done good work for many years, laboured under serious financial difficulties, and is one of the few undertakings of this nature which have not survived to the present day.

Several other dispensaries were founded between the dates of the rival Finsbury institutions. In 1782 the Eastern Dispensary was started to minister to the still unsatisfied needs of Whitechapel and Houndsditch. A new and interesting feature of this foundation was the formation of a kind of school for clinical practice in connection with it. The pupils of the physician went his rounds with him, and thus gained a degree of experience nowhere else to be obtained, since, as we have seen, the hospitals did not at that time offer a study of disease under anything like normal conditions. This educational aspect of the dispensary had been part of Dr. Lettsom's wisely thought-out scheme at the beginning, although it could not immediately be got into working order, and it became an important feature in most of the best dispensaries. The pupils were not allowed to prescribe for the patients, or perform any operation save under the immediate direction of the physician, and in his presence.

In 1783 the Public Dispensary was instituted for the benefit of the very crowded and poor district round St. Giles's, which has already been mentioned as one of the most desperate quarters of London, and included also besides, the districts of Holborn, Bloomsbury and several City parishes.

The St. Marylebone General Dispensary, founded two years later in Welbeck Street, took in part of the same district, viz., Bloomsbury, St. Giles's, and St. Martin's-in-the-Fields, in addition to the country parishes of Marylebone, St. Pancras, and Paddington.

Overlapping had now seriously begun. In 1789 Westminster added to its already numerous medical charities the Western Dispensary, situated in Charles Street. Although the promoters urged that from its situation it interfered with no other institution of the kind, it is certain there were three other dispensaries within half a mile of its doors. It was proposed to add gifts of "wine, sago, and invigorating nourishment" to the medical relief afforded, but it does not appear that funds were forthcoming to defray this expense.

Lastly must be mentioned the Tower Hamlets Dispensary, founded in 1792, under the ambitious title of the Universal Medical Institution. In addition to the ordinary purposes of a dispensary, the design was to provide cold, warm, and vapour baths for men employed in unhealthy trades, such as white lead manufactures. Special attention was given to recovery from drowning and suffocation. The institution was under the immediate patronage and direction of Dr. Lettsom, and his well-known partiality for cold water is indicated in the provision of "separate cold baths kept for the sole use of the governors, to bathe as often as agreeable free of expense."

